# The exceptions that prove the rule—a historical view of bedaquiline susceptibility

**DOI:** 10.1186/s13073-024-01311-w

**Published:** 2024-03-13

**Authors:** Paolo Miotto, Daniela M. Cirillo, Thomas Schön, Claudio U. Köser

**Affiliations:** 1 Emerging Bacterial Pathogens Unit, IRCCS San Raffaele Scientific Institute, Milan, Italy; 2 Department of Infectious Diseases, Region Östergötland, Linköping University Hospital, Linköping, Sweden; 3 Division of Infection and Inflammation, Institute of Biomedical and Clinical Sciences, Linköping University, Linköping, Sweden; 4 Department of Infectious Diseases, Kalmar County Hospital, Linköping University, Kalmar, Sweden; 5 Department of Genetics, University of Cambridge, Cambridge, UK

**Keywords:** *Mycobacterium tuberculosis*, Bedaquiline susceptibility, Resistance mutations, Clinical trials

## Abstract

In the accompanying study, Nimmo and colleagues estimated the dates of emergence of mutations in *mmpR5* (*Rv0678*), the most important resistance gene to the anti-tuberculosis drug bedaquiline, in over 3500 geographically diverse *Mycobacterium tuberculosis* genomes. This provided important insights to improve the design and analysis of clinical trials, as well as the World Health Organization catalogue of resistance mutations, the global reference for interpreting genotypic antimicrobial susceptibility testing results.

## Antimicrobial resistance predates the introduction of a drug

By the time smear-positive tuberculosis develops, the bacterial burden is high and, therefore, some bacilli will have developed resistance due to spontaneous mutations—even to antimicrobials that are not yet discovered. Considering this constantly replenishing pool of resistance prior to introduction of a drug, the question is to what extent these mutants can transmit and persist over time in the absence of antimicrobial pressure or additional mutations that compensate for potential adverse fitness effects [1]. Importantly, the older such mutants are, the more likely they are frequent and geographically widespread [1]. For example, *pncA* H57D, the mutation responsible for intrinsic resistance to the first-line drug pyrazinamide in most *Mycobacterium bovis* strains, is estimated to have evolved 900 years ago [1]. Its age notwithstanding, *M. bovis* disease rates in humans have fallen substantially in many countries because of milk pasteurisation and other measures (e.g. to < 2% in the Netherlands compared to about 10% in 1938 [2]). Intrinsic resistance to capreomycin predating the antibiotic era has also been reported in one *M. tuberculosis* subgroup, but this drug is no longer recommended for treating tuberculosis by the World Health Organization (WHO) [1].

## The bedaquiline era

Together with pretomanid, linezolid, and moxifloxacin, bedaquiline is now part of an all-oral 6-month regimen that is becoming the preferred option for treating rifampicin-resistant tuberculosis. The evidence is mounting that baseline bedaquiline resistance due to *mmpR5* (*Rv0678*) changes is likely a risk factor for failure of this regimen [3]. Moreover, some *mmpR5* resistance mutations predate the introduction of bedaquiline. The most important example is *mmpR5* Met146Thr, which evolved in the early 2000s and was recognised as a conferring cross-resistance to bedaquiline and clofazimine in the latest edition of the WHO catalogue of resistance mutations [4, 5]. Which, if any, selective pressure facilitated the spread of this mutation in Eswatini is unknown, but prior use of clofazimine is a potential factor as *mmpR5* encodes a negative regulator of the MmpS5-MmpL5 efflux pump that also exports clofazimine [4].

In the accompanying article, Nimmo et al. took a systematic approach to estimate the dates of emergence of *mmpR5* mutations in more than 3500 geographically diverse genomes of the *M. tuberculosis* lineages 2 and 4, which account for the majority of tuberculosis [6]. Where available, they used phenotypic antimicrobial susceptibility testing (AST) results to distinguish likely resistance mutations from those that are not. The findings from these analyses have immediate implications for the WHO mutation catalogue, which serves as the reference to interpret genotypic AST results, as well as clinical trials [5].

## Implications for WHO mutation catalogue

### Exceptions to loss-of-function (LoF) additional grading rule

Because *mmpR5* resistance mutations are rare in most settings, it is difficult to meet the thresholds employed by WHO to classify them as group 1 “associated with resistance” or group 2 “associated with resistance – interim” resistance mutations (both groups are interpreted as markers of bedaquiline/clofazimine cross-resistance) [5]. To improve the sensitivity of its catalogue, WHO endorsed an additional grading rule, whereby any LoF change in *mmpR5* that could not be classified based on experimental evidence should be assumed to confer a LoF phenotype and classified as group 2 bedaquiline/clofazimine resistance mutation. LoF changes were defined as full-gene deletions, frameshifts, mutations that abolish the start codon, and premature stop codons [5]. Although this additional grading rule was supported by the finding that pooled LoF mutations meet the WHO criteria for group 1 bedaquiline mutations, WHO acknowledged that exceptions may exist and that these should be investigated as a priority (e.g. a nonsense mutation that shortens a protein by one amino acid is unlikely to confer a LoF phenotype) [5].

Two studies suggest that a frameshift at codon 141, currently classified as a group 2 resistance mutation using the aforementioned rule, may not confer resistance to bedaquiline or clofazimine [7, 8]. Having emerged around 1870, Nimmo et al. estimated this frameshift to be the third-oldest mutation in their dataset, where it occurred in four genomes from Uganda and two from the UK [6]. This frameshift should be prioritised for further characterisation to establish whether it really is an exception to the LoF grading rule, in which it should be reclassified as a group 4/5 mutation that is not relevant for resistance to avoid systematically overcalling bedaquiline/clofazimine cross-resistance [5].

### Exceptions due to epistasis

Frameshifts at codon 67 of *mmpR5* were one of only five changes with sufficient high-quality AST data to be classified as group 1 resistance mutations by WHO [5]. Indeed, frameshifts at this codon arose repeatedly since the late 1990s, in line with selective pressure by antibiotic retreatment [6]. However, this frameshift also occurs in a cluster of predominantly rifampicin-resistant strains from Peru that they estimated to have evolved approximately 300 years ago [6, 9]. Notably, this cluster also harbours a *mmpL5* frameshift that counteracts the *mmpR5* frameshift. Mutations inactivating *mmpS5* likely also counteract resistance mutations in *mmpR5*, but because the dataset evaluated by WHO did not feature any such cases, WHO only endorsed that *mmpL5* needs to be considered when interpreting *mmpR5* mutations [5].

### Gaps in the catalogue

The WHO catalogue will have to be continuously updated given the large spectrum of existing and yet to be selected bedaquiline resistance mutations [5, 6]. As mentioned above, older mutations are more likely geographically widespread, which means that phenotypic AST capacity, ideally using high-quality MIC testing, should be directed to clarify whether other older mutations confer resistance or not. For instance, *mmpR5* Gly41Ala that likely arose between WW1 and WW2 could not be classified even using the criteria employed by Nimmo et al. that were usually less stringent than those adopted by WHO [5].

## Implications for clinical trials

### Exceptional hyper-susceptibility

C-11A *mmpR5*, which is mostly found in South Africa, arose about 180 years ago [6]. This mutation correlates with approximately fourfold lower bedaquiline MICs, whereas strains with an inactive MmpS5-MmpL5 efflux pump display more marked hyper-susceptibility to bedaquiline and clofazimine (the precise MIC ranges for these are not known as most studies did not test sufficiently low concentrations to obtain MIC endpoints [9]). Hyper-susceptibility due to LoF mutations in the efflux pump has evolved repeatedly throughout the *M. tuberculosis* phylogeny but appears to be rare globally [9]. Yet, if clinical trials are conducted in settings where such mutants are overrepresented, for example in Peru and South Africa, and this potential source of bias is not adequately considered, it might lead to misleading conclusions. Specifically, the hyper-susceptible strains may result in the efficacy of a regimen being over-estimated or even mask that the outcomes of strains that are not hyper-susceptible are unacceptably poor (i.e. that only hyper-susceptible strains have non-inferior outcomes compared to the standard of care). These factors must also be considered for trials of other antimicrobials that are exported by MmpS5-MmpL5 (e.g. BTZ-043, quabodepistat, and TBA-7371).

## Beyond bedaquiline and clofazimine

In our view, the approach by Nimmo et al. to systematically date the emergence of mutations in resistance genes should be extended to other novel antimicrobials and be carried out routinely as part of WHO surveillance studies (e.g. for pretomanid, for which lineage effects exist and to which intrinsic resistance is known to have emerged repeatedly [10]). Older mutations could be prioritised for characterisation to assess whether and how they affect the susceptibility to those agents, thereby improving clinical development and facilitating the development of phenotypic and genotypic AST assays.
